# Combining radiation therapy with immunotherapy: clinical translation

**DOI:** 10.1186/1479-5876-13-S1-K10

**Published:** 2015-01-15

**Authors:** Silvia C Formenti

**Affiliations:** 1 Department of Radiation Oncology and Medicine, New York University School of Medicine and New York University Perlmutter Cancer Institute, New York, New York, USA

## 

Ionizing radiation induces immunogenic cell death of tumors, an effect likely to contribute to the success associated with radiotherapy of cancer [1]. Recent discovery suggests that radiotherapy can be applied as a powerful adjuvant to immunotherapy and, in fact, can contribute to convert the irradiated tumor into an *in situ* vaccine, resulting in specific immunity against metastases [2]. Preclinical models of syngeneic tumors have reliably predicted clinical success, in distinct tumor settings and immunotherapy/radiation combinations [3-5]. As a first proof of principle trial, we translated the preclinical evidence of a successful combination with Flt3 ligand and RT [6] to a protocol of GM-CSF and RT, and demonstrated out of field objective responses in 27% of patients with multiple metastases of solid tumors, defined as an abscopal effect [7]. Parallel mechanistic studies in the lab in the syngeneic 4T1 mouse model of metastatic breast cancer demonstrated at intratravital microscopy that RT with anti-CTLA-4 increased the arrest of T cells in contact with tumor cells. The latter required interaction of NKG2D on CD8+ T cells with its ligand retinoic acid early inducible-1 (Rae-1) on the tumor cells, up-regulated by RT. Blocking NKG2D-Rae-1 interactions increased markedly the motility of anti-CTLA-4 treated T cells inhibiting their contact with irradiated tumor cells, and abrogated immune-mediated tumor rejection, suggesting a critical role of radiation-induced NKG2D ligands for the antitumor effects of anti-CTLA-4 [8]. In humans, a similar block of NKG2D is mediated by soluble MICA (sMICA), which is released by some tumors and reaches high levels in the serum [9]. Dranoff *et al* reported that in some patients sMICA levels dropped after initiation of Ipilimumab, due to the generation of anti-sMICA antibodies that led to its clearance [10]. Decreased levels of sMICA were associated with increased expression of NKG2D in T and NK cells, and corresponded to response to treatment. Anti-sMICA antibodies and sMICA levels can be measured in serum with ELISA by using recombinant MICA protein and anti-human MICA monoclonal antibodies [10]. Since RT is known to upregulate MICA on the surface of tumor cells [11] biopsies of tumors before and after radiotherapy and Ipilimumab could also be tested for expression of MICA.The preclinical success of the combination of anti-CTLA-4 antibody and RT was mirrored by abscopal responses in metastatic melanoma and NSCLC patients irradiated to one lesion, during Ipilimumab. This evidence inspired our current trial testing radiotherapy with CTLA-4 blockade in metastatic melanoma. In this study patients with newly diagnosed metastatic melanoma eligible to first line Ipilimumab are randomly assigned to Ipilimumab alone or Ipilimumab and radiotherapy to one metastatic lesion. Preliminary results in seven patients demonstrate feasibility of the combination, without additive toxicity. The novel role of radiotherapy as a powerful adjuvant to immunotherapy warrants more research to define the optimal immunotherapy/RT combinations: currently 35 trials of RT+immunotherapy are ongoing in USA.
